# Deconstructing heterogeneity in schizophrenia through language: a semi-automated linguistic analysis and data-driven clustering approach

**DOI:** 10.1038/s41537-022-00306-z

**Published:** 2022-11-29

**Authors:** Valentina Bambini, Federico Frau, Luca Bischetti, Federica Cuoco, Margherita Bechi, Mariachiara Buonocore, Giulia Agostoni, Ilaria Ferri, Jacopo Sapienza, Francesca Martini, Marco Spangaro, Giorgia Bigai, Federica Cocchi, Roberto Cavallaro, Marta Bosia

**Affiliations:** 1 Department of Humanities and Life Sciences, University School for Advanced Studies IUSS, Pavia, Italy; 2 Department of Clinical Neurosciences, IRCCS San Raffaele Scientific Institute, Milan, Italy; 3 School of Medicine, Vita-Salute San Raffaele University, Milan, Italy

**Keywords:** Schizophrenia, Human behaviour

## Abstract

Previous works highlighted the relevance of automated language analysis for predicting diagnosis in schizophrenia, but a deeper language-based data-driven investigation of the clinical heterogeneity through the illness course has been generally neglected. Here we used a semiautomated multidimensional linguistic analysis innovatively combined with a machine-driven clustering technique to characterize the speech of 67 individuals with schizophrenia. Clusters were then compared for psychopathological, cognitive, and functional characteristics. We identified two subgroups with distinctive linguistic profiles: one with higher fluency, lower lexical variety but greater use of psychological lexicon; the other with reduced fluency, greater lexical variety but reduced psychological lexicon. The former cluster was associated with lower symptoms and better quality of life, pointing to the existence of specific language profiles, which also show clinically meaningful differences. These findings highlight the importance of considering language disturbances in schizophrenia as multifaceted and approaching them in automated and data-driven ways.

## Introduction

Language disorders are a core feature of schizophrenia, with more than 70% of individuals showing linguistic and communicative impairments^1,2^. Disturbances might affect all levels of linguistic processing, from the “building blocks” of language, including speech characteristics, grammatical structures, and lexical components, up to more sophisticated aspects such as pragmatic interpretation^3^. In particular, schizophrenia has been associated with altered pausing and prosody, reduced grammatical processing skills, diminished lexical richness (e.g., lower type-token ratio), and defective semantic processes^4–8^. Studies reported also impairment in the ability to manage discourse and conversation, as well as to understand non-literal expressions^9–11^. These linguistic and communicative difficulties show extensive correlations with cognitive aspects^1,2^ and have been linked to both positive (in particular, formal thought disorder and disorganization) and negative (especially poverty of speech) symptoms^4,5,12,13^. Furthermore, language disturbances are associated with reduced daily functioning^1,14^. In particular, among the different domains of daily functioning, language and communication have been shown to impact especially community integration, interpersonal relations, and social functioning at large^15,16^. Taken together, this evidence makes language a relevant domain of assessment also for clinical purposes.

The last decades witnessed the rise of computational approaches to provide quick and fine-grained quantitative linguistic analysis. Although still not used on the large scale, these methods have already proven successful in different clinical applications on individuals with schizophrenia^17–19^. First, these approaches showed high accuracy levels in distinguishing individuals with schizophrenia from healthy controls^20,21^ and first-degree relatives^22^, as well as for differential diagnosis (e.g., schizophrenia vs. bipolar disorder)^23^. Furthermore, they were successfully applied also for prognostic purposes. In individuals at clinical high risk for psychosis, computational methods were able to predict conversion to psychosis with high levels of accuracy up to 100%^24–26^. Moreover, in individuals at first episode of psychosis, computational approaches were effective in predicting diagnostic outcome up to eighteen months^27,28^. Indeed, most of the current research in this domain focuses on at-risk individuals and in supporting diagnosis in first-episode psychosis, in line with the idea of linguistic impairment as a potential biomarker of schizophrenia^18,19^.

However, little research has examined via automated methods the linguistic characteristics exhibited by individuals with a long-term history of schizophrenia. Among the few studies documenting an application of computational methods in chronic schizophrenia, de Boer et al.^29,30^ showed that automatically extracted speech features (e.g., articulation rate, number and duration of pauses, and mean length of utterance) and lexical features (i.e., type-token ratio) were associated with negative symptoms as well as with the integrity of the white matter in the language tracts. Buck et al.^31^ used the Linguistic Inquiry and Word Count (LIWC)^32^ software to distinguish stories produced by patients vs. healthy controls, highlighting especially the correlations between the number of words, symptoms, and sociocognitive skills. Similarly, Minor et al.^33^ reported that altered use of emotional words extracted via LIWC was related to higher negative symptomatology and lower functioning in schizophrenia. In the same vein, other studies found that individuals with schizophrenia showed less words per sentence and increased use of self-reference pronouns compared to controls^21,34^, without a difference in lexical variety as measured with the type-token ratio^21^.

Notably, these studies focused on the associations between automatically-extracted linguistic features and participants’ psychopathological, functional, cognitive, or sociocognitive characteristics, while a deeper machine-driven investigation of the heterogeneity of the linguistic profile of chronic patients has been generally neglected. This is a limitation since individuals with a long history of schizophrenia are greatly heterogenous under several respects^35–37^. For instance, different machine-learning clustering techniques have been successfully applied to decompose the psychopathological heterogeneity of chronic schizophrenia into sub-groups of individuals characterized by different patterns of negative symptomatology^38,39^. Similarly, clustering algorithms have proven effective in identifying clusters of participants with distinct levels of severity in cognitive and sociocognitive deficits^40–42^. Importantly, these approaches showed that clusters of individuals with different psychopathological and cognitive profiles might have different functional outcomes, as well as different responses to treatment^38,43,44^. More recently, also speech features were used to automatically identify clusters of patients with different levels of language impairment, which in turn were associated with distinct profiles of cognitive dysfunction^45^. In this light, data-driven approaches combined with automatically-extracted linguistic features might be key to refine the identification of subtypes of individuals with schizophrenia and obtain more nuanced and sensitive information about psychopathology and its nature, as well as about treatment responsiveness in the long-term illness progression^20^.

The present study aimed at extending the application of semi-automated linguistic approaches to unravel the clinical heterogeneity of schizophrenia, by innovatively combining computational linguistic methods with data-driven clustering techniques. Specifically, the study objectives were: (i) to identify separate subgroups of individuals with chronic schizophrenia based on a set of (semi-)automatically-extracted linguistic features, targeting the core “building blocks” of the language faculty, and (ii) to test whether the obtained linguistic subtypes are associated with differences at the psychopathological, daily functioning, cognitive, and sociocognitive levels.

To do so, we first developed a multilevel semi-automated linguistic analysis that took into account different linguistic domains described as compromised in schizophrenia^4,5,29,31,33^, from speech to the lexicon, and then applied a machine-learning unsupervised algorithm to the output variables of the linguistic analysis to identify clusters of participants characterized by distinct linguistic profiles. Clusters were finally compared for psychopathological, cognitive, sociocognitive, and functional aspects. We expected to find evidence of distinct linguistic profiles, and that these might be indicative of underlying psychopathological, cognitive, sociocognitive, and functional differences, consistently with previous studies that emphasised a relationship between linguistic impairment and clinical and functional measures^14,29,33^ and between language and cognitive and sociocognitive aspects^1,31,33^.

## Results

### Sample description

The sample included 67 participants, which were 26 females and 41 males, with a mean age of 39.75 ± 11.04 years and a mean education of 11.94 ± 2.72 years. The mean illness duration was 15.60 ± 10.70 years, with a mean age of onset of 24.28 ± 6.36 years. All participants were treated with antipsychotic therapy for at least 3 months (atypical/typical antipsychotic treatment: 61/6), with a mean Chlorpromazine-equivalent dose of 440.98 ± 200.57 mg/d.

### Overview

**Fig. 1:**
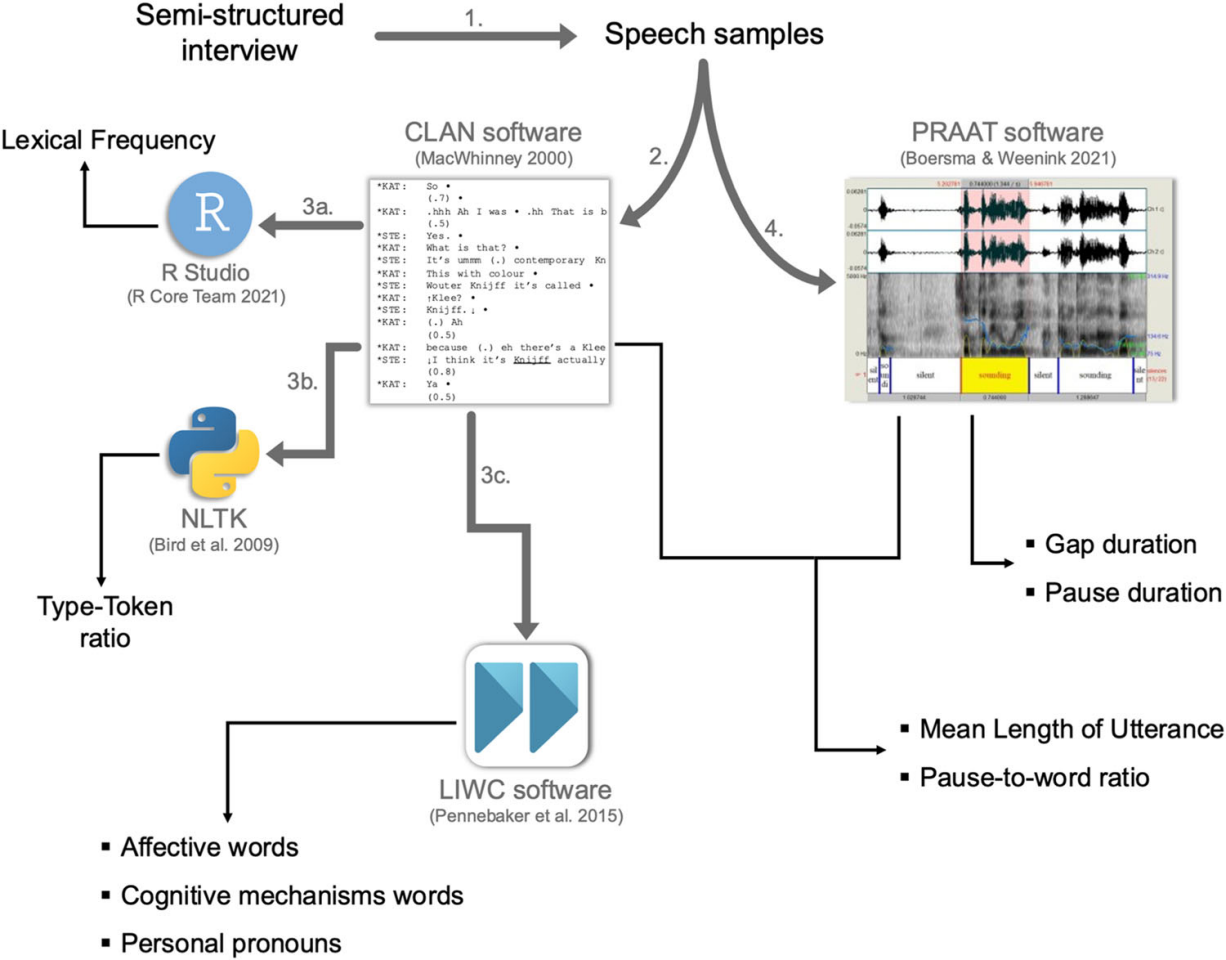
Visual representation of the pre-processing of audio files and extraction of the linguistic measures. Speech samples were obtained via the semi-structured interviews of the APACS test (1), and then transcribed using the CLAN software (2). Afterwards, token-based values were automatically extracted from the transcripts: R Studio was used to automatically obtain lexical frequency values for each token in the text from the Corpus and Frequency Lexicon of Written Italian (CoLFIS) corpus (3a), Natural Language Toolkit (NTLK) was employed to compute the Type-Token ratio (3b), while the Linguistic Inquiry and Word Count (LIWC) software was used to obtain the frequency of affective words and words indicating cognitive mechanisms (i.e., Psychological Lexicon) and Personal Pronouns (3c). Finally, the speech samples were processed using the PRAAT software (4) to determine the number of utterances for the computation of the Mean Length of Utterance, as well as to extract pause and gap duration and the number of pauses, used for the computation of the Pause-to-word ratio.

The linguistic features extracted via the semi-automated analysis targeted the following levels: (1) speech, (2) lexical richness, (3) occurrence of specific part-of-speech categories, in particular personal pronouns, and (4) occurrence of selected semantic classes, in particular within the psychological lexicon, as described in details in Table 1. A visual representation of audio file pre-processing and linguistic features extraction is provided in Fig. 1. The results of the analysis are presented in the following three sections, consistently with the three stages of the analysis: (i) the Principal Component Analysis (PCA), used to reduce data dimensionality and to identify Principal Components (PCs) emerging from the automatically extracted linguistic features, namely a smaller number of uncorrelated linguistic-based variables from the larger set of data; (ii) the cluster analysis, which used the linguistic-related variables obtained from the PCA to identify distinct clusters (i.e., sub-groups) of participants, characterized by high intra-class similarity and low inter-class similarity in their linguistic profiles; (iii) the comparison of the sub-groups resulting from the clustering process for clinical, neurocognitive, sociocognitive, and functional characteristics.

**Table 1:** Description of the linguistic features in the multilevel semi-automated analysis.

Linguistic dimensions	Measures	Description
Lexical Richness	Type-token ratio	The number of unique words divided by the total number of words in the speech sample; this measure is considered as an indicator of lexical variety and might reflect language or thought disorder^5,86^.
	Lexical frequency	Mean frequency value of all uttered words, obtained from the Corpus and Frequency Lexicon of Written Italian (CoLFIS)^78^; it indicates whether the participant used more low- or high-frequency words.
Fluency	Mean length of utterance (in words)	Total number of words produced in each utterance divided by the total number of utterances; this measure might reflect poverty of speech.
	Mean gap duration (in msec)	Total duration of silences between the interviewer’s question and the participant’s answer (gap) divided by the total number of gaps; this value reflects average turn planning time^87^.
	Mean silent and filled pause duration (in msec)	Total duration of silent pauses (defined as silences longer than 200 msec) and filled pauses (e.g., *uhm*, *ehm*, etc.) divided by the total number of pauses; pause duration reflects intra-turn planning and self-monitoring processes^29^.
	Pause-to-word ratio	Total number of pauses divided by the total number of words in the speech sample; this value can be considered as an indicator of processing speed^29^.
Frequency of Personal Pronouns		Percentage of personal pronouns over the total word count.
Psychological Lexicon	Frequency of affective words	Percentage of words conveying positive or negative emotional valence over the total word count.
	Frequency of words related to cognitive mechanisms	Percentage of words expressing causality, insight, possibility, inhibition, or certainty (e.g., *because*, *hence*, *think*, *know*, *consider*, *ought*, *should*, *exclude*, etc.) over the total word count. This measure might reflect metacognitive processes^48^.

#### Principal Component Analysis on linguistic features

**Fig. 2:**
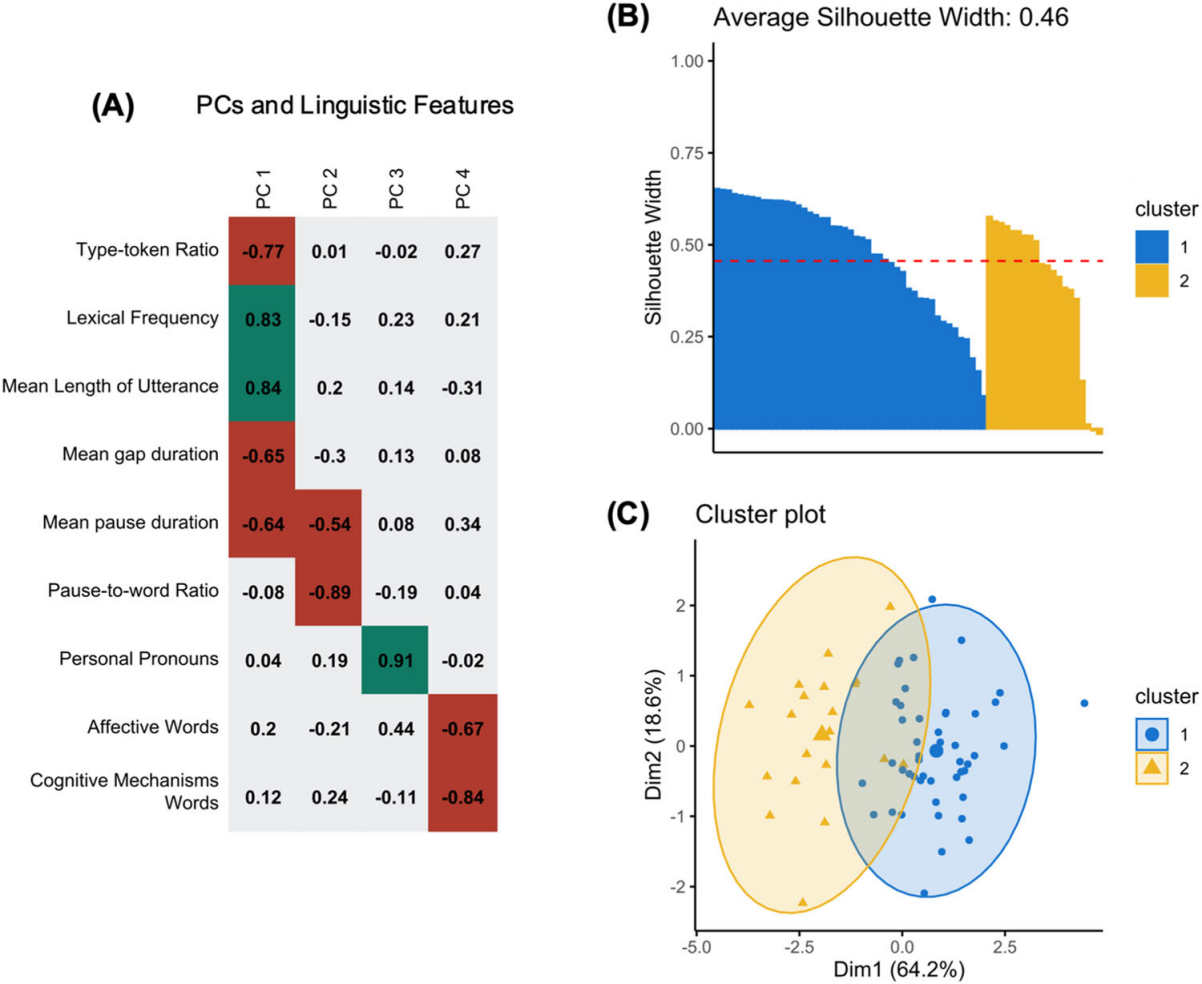
Results of the principal component analysis and cluster analysis. **A** Associations between the four principal components (PCs) identified by the Principal Component Analysis and the linguistic features; green-colored boxes indicate a positive association, while red-colored boxes a negative association. **B** Silhouette width for participants included in both clusters (horizontal axis) and average silhouette width for the two-cluster solution (red dashed line). **C** Clusters distribution around centroids.

The PCA performed on the linguistic features identified *n* = 4 meaningful PCs (with eigenvalue >1), describing respectively 39.80%, 13.52%, 12.23%, and 11.30%, for a total of 76.85% of data variance. To better characterize the unique contribution of the linguistic features on each PC, we considered only high factor loadings (|>0.50|)^46^. Lexical Richness features (type-token ratio and lexical frequency) and specific Fluency features (i.e., mean length of utterance, mean gap duration, and mean pause duration), reflecting lexical access and speech planning processes, loaded primarily on PC1. Fluency characteristics related to pauses (i.e., mean pause duration and pause-to-word ratio) loaded also on PC2, which intercepts speech planning and monitoring and speed processing. Frequency of Personal Pronouns loaded exclusively on PC3, while frequency of Psychological Lexicon (i.e., affective and cognitive mechanisms words) loaded selectively on PC4. The correlations between the linguistic features and each PC are shown in Fig. 2A.

#### Cluster Analysis on linguistic-based PCs

The four linguistic-based PCs resulting from the PCA were used to run a cluster analysis using a k-means algorithm. The k-means algorithm identified two distinct clusters (see Supplementary Table 1 for the average silhouette width of different solutions). Figure 2B, C shows the silhouette profile of the two-cluster solution and participants’ distribution between clusters, respectively.

Participants were grouped by the k-means algorithm considering whether they showed higher (↑) or lower (↓) performance across the PCs associated with the different linguistic domains. The algorithm assigned participants to Cluster 1 (*n* = 47) if their speech was characterized by ↓Lexical Richness, ↑Fluency, ↑Pronouns, and ↑Psychological Lexicon. Conversely, participants were assigned to Cluster 2 (*n* = 20) if their speech was characterized by ↑Lexical Richness, ↓Fluency, ↓Pronouns, and ↓Psychological Lexicon. Clusters’ centroids are reported in Supplementary Table 2. Examples of participants for each cluster (i.e., excerpts of the transcripts of their interviews and individual data) are reported in Supplementary Tables 4, 5.

#### Validation of the cluster analysis

The optimal two-cluster solution identified through the k-means algorithm was validated using an independent algorithm by performing a Linear Discriminant Analysis (LDA) with two approaches: (a) a random-split samples procedure (to assess the degree of classification concordance over *n* = 50 iterations across different partitions), (b) a leave-one-out method.

**Fig. 3:**
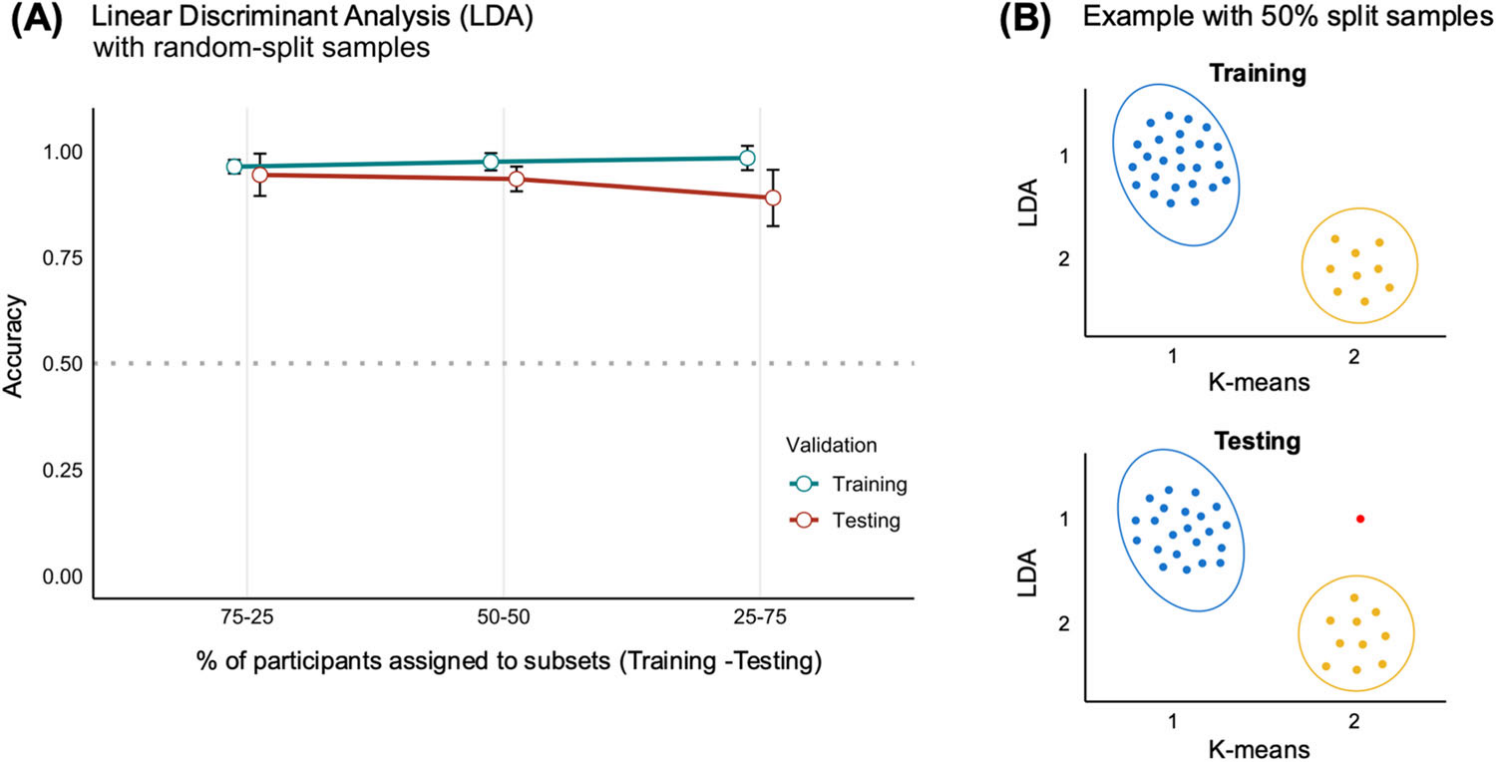
Results of the linear discriminant analysis (LDA) with random-split samples. **A** Mean values of training and testing accuracy (error bars indicate standard deviations), computed on random samples with 75%, 50%, and 25% of participants of the original sample assigned to the training subset and the remaining to the testing subset (50 iterations performed using the same method). The general performance of the classification function remains high and stable across different training-testing partitions. **B** Conceptual representation of a replication with 50% of participants randomly assigned to the training subset and the other 50% to the testing subset (training accuracy: 100%; testing accuracy: 97%). The outcome of this single replication shows that in the testing subset only one participant from Cluster 2 is misclassified by the model.

As for the LDA with random-split samples procedure, with 75% of participants assigned to the training set, the mean training accuracy was 0.96 ± 0.02 and the mean testing accuracy was 0.95 ± 0.05; with 50% of participants randomly assigned to the training set, the mean training accuracy was 0.97 ± 0.02 and the mean testing accuracy was 0.93 ± 0.03; finally, with 25% of participants assigned to training, the mean training accuracy was 0.98 ± 0.03 against a mean testing accuracy of 0.89 ± 0.07. Overall, the algorithm showed a stable performance across training-testing partitions (see Fig. 3A; see Fig. 3B for a conceptual representation of the results of one replication with a 50% training-testing partition).

The LDA with leave-one-out cross-validation confirmed the random-split samples procedure, highlighting an almost perfect level of agreement with the cluster solution identified through the k-means algorithm (with 94% of participants correctly assigned to the two clusters, see Supplementary Table 3).

Taken together, these validation methods support the stability of the two-cluster solution over several repetitions.

#### Cluster comparison

Since all dependent variables were normally distributed and had homogeneous variance, we proceeded to perform a series of *t*-tests to compare the two clusters. Participants in Cluster 1 and 2 did not differ for age, education, disease duration, age of onset, Chlorpromazine-equivalent dose, type of antipsychotic treatment, neurocognition as evaluated with the Brief Assessment of Cognition in Schizophrenia (BACS), or social cognition as evaluated with the Theory of Mind Picture Sequencing Task (ToM PST) (|*t*s|≤ 1.66; *p*s ≥ 0.102). Conversely, the two clusters were significantly different in psychopathology as evaluated with the Positive and Negative Syndrome Scale for Schizophrenia (PANSS) and in daily functioning as evaluated with the Quality of Life Scale (QLS) (Table 2).

**Table 2:** Demographic, clinical, cognitive, and functional descriptive measures of participants classified in Cluster 1 and Cluster 2, and results from *t*-test comparisons.

Measures	Cluster 1	Cluster 2	Test statistics	*p*-value
Age	40.62 ± 10.99	37.70 ± 11.16	*t*(65) = 0.99	0.326
Education	11.87 ± 2.82	12.10 ± 2.53	*t*(65) = −0.31	0.756
Illness Duration	15.91 ± 10.67	14.85 ± 11.00	*t*(65) = 0.37	0.712
Age of onset	24.68 ± 6.54	23.35 ± 5.98	*t*(65) = 0.78	0.438
Treatment (atypical/typical)	42/5	19/1	–	0.660^a^
Chlorpromazine-equivalent dose (mg/d)	414.79 ± 187.64	502.55 ± 220.93	*t*(65) = −1.66	0.102
BACS*	1.57 ± 0.87	1.52 ± 0.99	*t*(64) = 0.22	0.830
ToM PST Total	44.86 ± 11.52	44.53 ± 13.28	*t*(61) = 0.10	0.929
Quality of Life Scale IRe	20.91 ± 6.08	14.40 ± 5.83	*t*(64) = 4.05	<0.001
Quality of Life Scale IRo	4.83 ± 5.45	2.85 ± 4.94	*t*(64) = 1.39	0.169
Quality of Life Scale PA	28.96 ± 7.04	18.80 ± 8.01	*t*(64) = 5.17	<0.001
Quality of Life Scale Total	54.70 ± 14.08	36.05 ± 14.42	*t*(64) = 4.91	<0.001
PANSS Positive Scale	16.23 ± 3.76	18.70 ± 4.52	*t*(65) = −2.31	0.024
PANSS Negative Scale	19.72 ± 4.71	23.50 ± 3.95	*t*(65) = −3.14	0.010
PANSS General Scale	37.15 ± 6.62	41.55 ± 4.94	*t*(65) = −2.67	0.019
PANSS Disorganization	20.23 ± 5.10	23.45 ± 4.19	*t*(65) = −2.49	0.021

Degrees of freedom in the *t*-tests vary due to missing values on some tests. *P*-values referring to Quality of Life Scale and PANSS Scale are FDR adjusted.

*BACS* Brief Assessment of Cognition in Schizophrenia, *ToM PST* Theory of Mind Picture Sequencing Task, *IRe* Interpersonal Relations, *IRo* Instrumental Role, *PA* Personal Autonomy, *PANSS* Positive and Negative Syndrome Scale.

*Equivalent total score obtained from all subtasks in the BACS.

^a^This value refers to Fisher’s Exact Test.

**Fig. 4:**
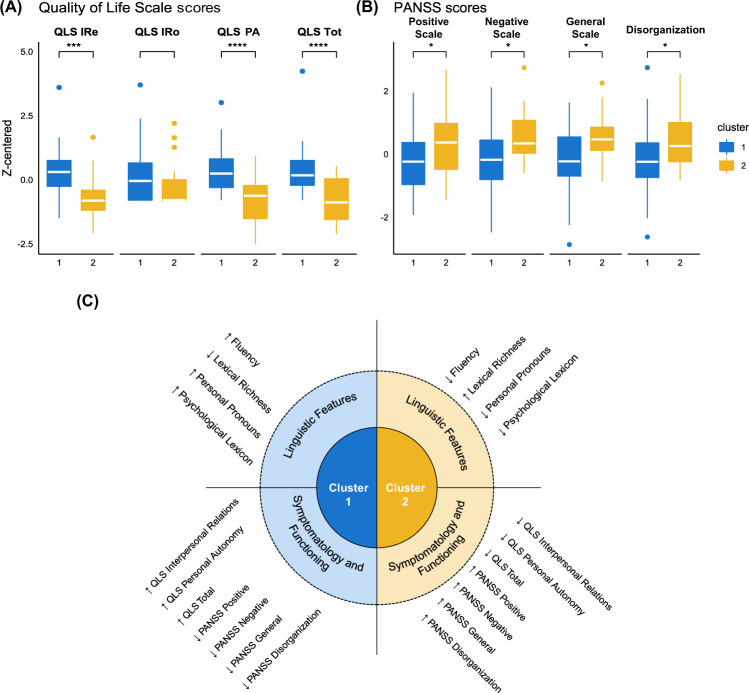
Results of cluster comparisons and summary of clusters. **A** Between-cluster comparisons for Quality of Life Scale (QLS), including Interpersonal Relations (IRe), Instrumental Role (IRo), and Personal Autonomy (PA) sub-scales and total score (Tot). **B** Between-cluster comparisons for PANSS scores (Positive, Negative, and General Scales and Disorganization dimension score). **C** Summary of the linguistic, psychopathological, and functional differences of the participants belonging to Cluster 1 and Cluster 2 (only significant differences are reported): arrows indicate higher (↑) or lower (↓) linguistic performance, psychopathological symptoms (as evaluated by the scores obtained in the PANSS Positive, Negative, and General scales and Disorganization score), and functioning (as evaluated with the QLS subscales and Total score).

In particular, participants in Cluster 1 exhibited higher QLS scores in the Interpersonal Relations (*t*(64) = 4.05; *p* < 0.001) and Personal Autonomy (*t*(64) = 5.17; *p* < 0.001) subscales, and in the Total (*t*(64) = 4.91; *p* < 0.001) score, even though they were not significantly different from participants in Cluster 2 in the Instrumental Role subscale (*t*(64) = 1.39; *p* = 0.169) (Fig. 4A). Moreover, participants in Cluster 1 exhibited a less severe symptomatology, as indicated by lower scores in the PANSS Positive (*t*(65) = −2.31; *p* = 0.024), Negative (*t*(65) = −3.14; *p* = 0.010), and General (*t*(65) = −2.67; *p* = 0.019) scales compared to Cluster 2, as well as by a lower score in the Disorganization dimension (*t*(65) = −2.49; *p* = 0.021) (Fig. 4B). A comprehensive summary of the linguistic, functional, and psychopathological profile of the two clusters is provided in Fig. 4C.

#### Additional analysis on cognition and social cognition

We furtherly compared clusters across the subtasks of the BACS and the ToM PST, in order to investigate possible differences in cognition and social cognition between the two subgroups in a more fine-grained fashion. The *t*-tests revealed that the two clusters did not differ for BACS and ToM PST subscores (|*t*s|≤ 1.38; *p*s ≥ 0.172) (Table 3).

**Table 3:** Descriptive measures of BACS and ToM PST subtasks for participants classified in Cluster 1 and Cluster 2, and results from *t*-test comparisons.

Measures	Cluster 1	Cluster 2	Test statistics	*p*-value
BACS Verbal Memory	45.79 ± 10.10	41.90 ± 11.32	*t*(64) = 1.38	0.172
BACS Digit Sequencing	17.97 ± 4.49	17.09 ± 5.82	*t*(64) = 0.67	0.506
BACS Token Task	33.74 ± 9.37	35.20 ± 7.24	*t*(61) = −0.59	0.555
BACS Semantic Fluency	23.76 ± 16.34	19.63 ± 17.45	*t*(63) = 0.92	0.360
BACS Symbol Coding	40.71 ± 12.06	40.16 ± 14.29	*t*(63) = 0.16	0.876
BACS Tower of London	14.60 ± 3.64	13.85 ± 4.54	*t*(65) = 0.71	0.483
ToM PST Sequencing	27.63 ± 7.72	27.00 ± 9.04	*t*(61) = 0.28	0.778
ToM PST Questionnaire	17.30 ± 4.62	17.53 ± 4.43	*t*(61) = −0.18	0.855

BACS subscores are adjusted for age and education. Degrees of freedom in the *t*-tests vary due to missing values in some subtasks.

*BACS* Brief Assessment of Cognition in Schizophrenia, *ToM PST* Theory of Mind Picture Sequencing Task.

**Fig. 5:**
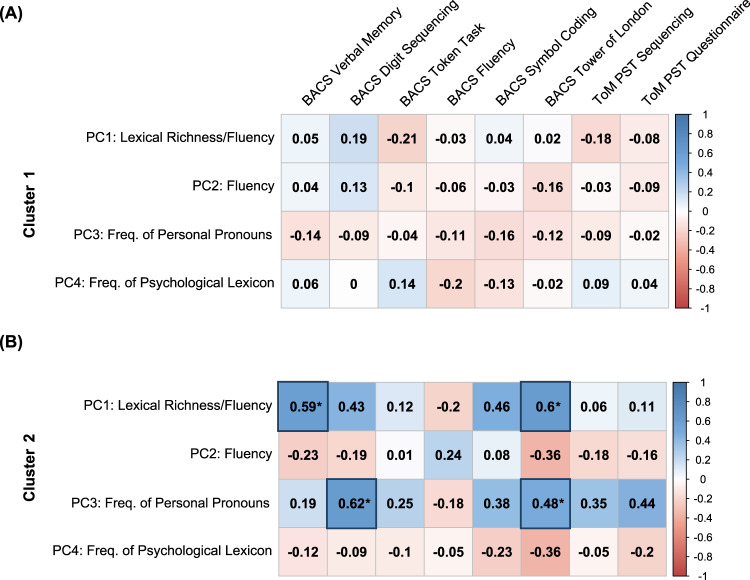
Results of the correlation analysis with BACS and ToM PST subscores across clusters. **A** Correlations between linguistic-based principal components (PCs) and BACS and ToM PST subscores for Cluster 1. **B** Correlations between linguistic-based PCs and BACS and ToM PST subscores for Cluster 2 (significant correlations are indicated with the asterisk, with significance level *p* < 0.05).

Then, we exploratively assessed the correlation between the BACS and ToM PST subscores on the one hand and the linguistic-based PCs on the other hand in each cluster separately, to assess patterns of association between participants’ linguistic profile and the other cognitive domains. No significant associations were observed between the linguistic PCs and the cognitive and sociocognitive measures for participants in Cluster 1 (|*r*s|≤ .21, *p*s ≥ 0.185) (Fig. 5A). Conversely, Cluster 2 exhibited an overall stronger pattern of correlations between linguistic and cognitive aspects. In particular, the correlations between PC1 scores (i.e., Fluency/Lexical Richness) and BACS Verbal Memory and Tower of London subscores, and between PC3 scores (i.e., Frequency of Personal Pronouns) and BACS Digit Sequencing and Tower of London subscores (Fig. 5B) reached significance. No other robust significant associations between the linguistic-based PCs and the ToM PST subscores were found in Cluster 2.

These data indicate that, although the distribution of cognitive and sociocognitive scores was not different across clusters, in Clusters 2 participants’ the linguistic profile was linked to a greater extent with cognitive characteristics, while in Cluster 1 linguistic and cognitive variables seem to be relatively independent.

## Discussion

In this study, we used an innovative multidimensional semi-automated linguistic analysis combined with a data-driven clustering algorithm to identify different linguistic profiles in individuals with schizophrenia and test differences at psychopathological, cognitive, sociocognitive, and functional levels. This method identified two clusters of participants based on their linguistic features, which – in line with our expectations – turned out to show also different patterns of psychopathological, functional, and cognitive characteristics. On the one hand, Cluster 1 – characterized by higher Fluency, higher Frequency of Personal Pronouns, lower Lexical Richness but higher Psychological Lexicon – was associated with lower psychopathological symptomatology and better quality of life; on the other hand, Cluster 2 – characterized by lower Fluency, lower Frequency of Personal Pronouns, higher Lexical Richness but lower Psychological Lexicon – was associated with higher psychopathological symptomatology and worse quality of life. Moreover, relative to Cluster 1, the linguistic profile of participants belonging to Cluster 2 showed a stronger pattern of correlations with the underlying cognitive skills, with lower linguistic scores being associated with poorer cognitive functioning. These findings open up at least three different orders of considerations, concerning (i) the importance of multidimensional language assessment; (ii) the specific associations between linguistic profiles and psychopathological, functional, and cognitive domains; and (iii) the clinical implications and possible developments of this type of approach.

Concerning language assessment, the first aspect to notice is that the linguistic profiles emerged from a multilevel language analysis, spanning from speech characteristics to the occurrences of words in specific semantic classes. The PCA identified four meaningful components that targeted different dimensions, and all of them fed the clustering algorithm, indicating great intergroup variation on all four extracted components, as well as high within group homogeneity. The high number of characteristics which define the linguistic profiles of our participants supports the idea that language and communicative impairments in schizophrenia cover a wide range of phenomena and represent an important source of heterogeneity among individuals^3,35^.

To examine more in depth the outcome of the linguistic analysis, it is important to comment on the specific clusters of linguistic features that emerged from the automatic classification. Participants belonging to Cluster 1 showed longer utterances and shorter and less numerous pauses and fillers; consistently, functional words such as pronouns were also more numerous; despite the greater fluency, words were globally more common and less various (as indicated by the higher lexical frequency and the lower type-token ratio), yet the occurrence of specific words referring to emotional content or cognitive processes was higher. In short, this cluster includes individuals who are more fluent in their speech (and possibly verbose) and use more psychological terms, but overall exhibit lower lexical variety. The linguistic profile of Cluster 1 confirms previous descriptions of altered type-token ratio^5^ and sparse evidence of redundancy (i.e., overuse of the same highly frequent words)^47^ in schizophrenia, which might occur also in highly fluent individuals. Conversely, speech samples acquired from participants belonging to Cluster 2 were characterized by shorter utterances, longer and more frequent pauses and fillers, lower occurrence of pronouns and emotional and metacognitive words, but in the context of higher variety and rarity (i.e., low frequency) of the terms used. In short, this cluster includes individuals who are less fluent and use less pronouns and psychological terms, but overall exhibit a greater lexical variety. The characterization of this cluster confirms the evidence of diminished fluency and the presence of altered use of pronouns and emotional words in schizophrenia^4,29,33,48^. What is important to highlight is that, while the various aspects that characterize each cluster were already noted in the literature as domains of impairment in schizophrenia, our analysis unravels distinct ways in which these domains of impairment might cluster, rather than simply co-occur. Compared to previous studies that entered unitary language scores, we were able to reveal a novel separation of clusters across a set of different linguistic features. Importantly, Cluster 1 and Cluster 2 should not be interpreted as two subgroups with high and low severity of language impairment, but rather as different profiles where language difficulties pattern differently, resulting in very distinctive speech characteristics. The direction of the association between fluency and lexical richness (measured as type-token ratio) in the two clusters is particularly interesting. While at first sight the fact that more fluent participants exhibited also lower lexical variety might appear counterintuitive, speech excerpts from our sample (see Supplementary Tables 4, 5) clearly illustrate that more talkative individuals can be more repetitive and redundant at the lexical level, which results in a lower type-token ratio, and that by contrast less fluent individuals might use more rare and unusual terms, resulting in a higher type-token ratio. In line with this, recent literature reported that, compared to healthy controls, individuals with schizophrenia have reduced fluency (e.g., shorter utterances and longer pauses) as well as higher lexical variety in terms of type-token ratio^29,30^. Our data confirm this evidence and add also that participants’ global lexical richness in terms of unique lemmas might be dissociated from the frequency of use of words belonging to specific semantic classes (e.g., psychological lexicon), with these two dimensions capturing different aspects of language impairment. Indeed, participants in Cluster 2 exhibited lower lexical variety but greater use of affective or metacognitive words, whereas individuals in Cluster 1 were poorer in the psychological lexicon, despite greater lexical richness.

The most relevant aspects distinguishing the two clusters are their different associations with psychopathological and functional domains, as they emerged from the step 3 of the analysis. This is overall the most innovative finding of our work and deserves special considerations for its clinical implications. Individuals with the more talkative yet lexically poorer profile (Cluster 1) exhibited lower symptoms and better functioning compared with individuals with the opposite profile (Cluster 2). On the one hand, these patterns are in line with associations previously reported in the literature, in particular the well-documented link between reduced fluency (longer pauses and shorter utterances) and negative symptoms^4,30^ and between speech and language impairment at large and disorganization aspects, such as formal thought disorder^5,49,50^ and difficulty in abstract thinking^10,11,51^. Our data also strengthen the connection between worse quality of life and linguistic aspects such as poverty of speech and greater use of (especially negative) emotional words^14,31,33^. On the other hand, our results markedly expand the state of the art by showing that a multilevel linguistic profiling might evidence different subgroups of individuals who, in addition to speaking differently, are also different not just in one psychopathological dimension but in the overall clinical severity. Going more deeply in this, it is interesting to note that those individuals who are more fluent, although possibly repetitive, are also those with greater use of mental state terms: this might be indicative of a greater attention to interpersonal relations and effort to engage in social interaction and conversation on personal topics, consistently with the better functioning observed for this subgroup (including the Interpersonal Relations subscale of the QLS). Conversely, the poverty of speech and reduced use of psychological lexicon as observed in Cluster 2 might reflect social withdrawal and poorer emotional self-awareness, consistently with the lower functioning and higher symptomatology of this subgroup. In sum, it seems that it is not just the amount of speech produced, or just the quality of the words, that reflects the clinical state, but rather it is the global linguistic profile, from speech to the lexicon, that is associated with different clinical outcomes.

However, the two clusters did not show any straightforward difference in cognition and social cognition, namely the two clusters did not vary in the global cognitive score and in the ToM score. While this finding is at first sight unexpected, the non-significant difference between groups can be explained when considering that the two clusters are not to be intended as reflecting different levels of severity in language impairment but rather as different linguistic profiles. While previous studies that evidenced a link between language and cognition adopted a whole-group correlational approach^1,2^, here the two clusters reflect different configurations of language characteristics, which seem to be relatively independent of the degree of cognitive impairment. Importantly, when we performed an additional fine-grained correlational analysis, different patterns of associations between linguistic features and cognitive aspects emerged for the two clusters. Participants in Cluster 2 showed significant correlations between Fluency/Lexical Richness and both verbal memory and planning abilities, as well as between the Frequency of Personal Pronouns and working memory and planning skills, while this pattern was not observed in Cluster 1. The stronger link between language and cognitive skills observed in Cluster 2 seems to suggest that, in individuals with more severe symptoms and poorer functioning, language and cognition are mutually dependent and linguistic impairment goes hand in hand with cognitive deterioration. Conversely, in more preserved individuals language and cognitive abilities seem to be rather independent. This additional analysis clarifies that our findings are not in contrast with the literature that found a relationship between linguistic abilities and the cognitive domains based on whole-group analysis^1,2^, adding that the link between language and cognition might have different strengths in different subgroups of patients. As for social cognition, no significant associations were highlighted in the additional analysis. When looking at the literature, social cognition seems to be a robust determinant of high-level (e.g., pragmatic) language skills in schizophrenia^1,2,15^, but the association with the building blocks of language in the domains of speech and lexical features is less robust and often not found^31,48,52^. Our data align with such evidence of a relative interdependence between language profiling and sociocognitive skills. It should also be noted, however, that in this study sociocognitive assessment was based on a single test focusing on mentalizing skills, leaving out other facets of the sociocognitive domain (e.g., emotion recognition) that might be important for language.

Finally, a comment is needed on the absence of difference between clusters in terms of demographic variables and characteristics such as onset and duration of illness. While acknowledging that these factors often affect the severity of clinical outcome^53,54^, the literature reports also evidence of clusters based on linguistic and cognitive characteristics with no variation in illness duration and onset^38,44,55^ or in education^38,44,45^. As for our data, the sample was characterized by chronic patients with an adult onset (ranging mostly between 20 and 30 years), which might further motivate the absence of differences between the two clusters. Similarly, education was homogenously distributed in the sample (with most participants having 8–13 years of education), hence with little room for variation. We cannot exclude, however, that in a larger and more diverse sample, linguistic profiling might capture also differences in education and illness duration and onset. Taking all these considerations together, our results provide novel evidence on how language features might combine in different multidimensional profiles, highlighting in particular a more fluent yet repetitive subgroup and a less fluent and poor in mental terms type, with the latter associated with worse clinical outcome. The cluster analysis that we presented underwent an articulated validation procedure suggesting that the classification scheme is rather stable, but certainly further studies are needed to validate it with independent samples.

The last important point to discuss focuses precisely on the linguistic-driven cluster analysis. Here we tried to extend the potential of computational methods, by innovatively combining them with a data-driven clustering technique as used to subtyping schizophrenia along other dimensions such as symptoms and cognitive functions^38,39,44^. The outcome of this approach contributes to corroborating the view that automated linguistic approaches in schizophrenia are much more than “promising”^56^, and could be of great utility not simply to predict diagnosis but also to monitor and deconstruct clinical heterogeneity throughout the illness course. Their combination with data-driven clustering approaches, as implemented in this study, could actually contribute greatly to unraveling subgroups of individuals with different clinical characteristics starting from the building blocks of language. Given the multifaceted nature of language impairment, the automatic language- and data-driven approach could not only overcome the subjective biases of the assessor but also disclose a greater amount of variability embedded in the linguistic data^57^ and is hence particularly suited to describe the vastly heterogenous population of individuals diagnosed with schizophrenia.

The results obtained with this method suggest a number of possible clinical implications. First, they provide the possibility of better orienting treatment strategies according to the individual’s global linguistic profile, following theoretically motivated, personalized, and multilevel approaches. In particular, adopting the multidimensional analysis to unravel the individual’s linguistic profile could allow to go beyond the clinical evaluation of language impairment and the simple observation of a fluent vs. non-fluent distinction to take into account also more subtle problems in the domains of speech and the lexicon that might escape the routine assessment. For instance, individuals with a profile of marked fluency in discourse associated with reduced lexical richness could benefit from treatment programs targeting verbosity and, when present, speech disorganization, coupled with exercises aiming at improving lexical breadth. Conversely, individuals with reduced fluency and limited use of pronouns and psychological lexicon might benefit from the combination of activities promoting verbal initiative and remediating under-informativity and exercises prompting the use of referential expressions as well as words expressing emotional states and metacognitive processes. Although the field of language rehabilitation in schizophrenia is at its infancy, there are already some effective programs available, improving different language components, such as for instance semantic classes and fluency features^58,59^, or discourse and pragmatics^60–62^, which could be selected and combined in a personalized fashion to increase treatment efficacy.

Additionally, our results suggest that automated assessments of linguistic characteristics and patterns, if implemented in clinical practice, may represent a useful tool to monitor treatment response and clinical course. Indeed, automatically detected changes in speech patterns may be associated and even precede changes in the clinical status. Going even further, this approach may be exploited as potential endpoints for the evaluation of clinical trials^57^, providing a more objective, comparable, and operator-independent measure of psychopathological and functional outcomes.

Nonetheless, what we proposed is just a first step towards a more integrated automated and machine-learning approach to language in schizophrenia. There are indeed several limitations in our study, which also disclose possible challenges for future research. First, although we conducted a solid machine-driven validation of the cluster analysis, future studies are needed to test the replicability of the linguistic grouping proposed here on independent samples. To increase reproducibility, a more massive machine-learning approach with the inclusion of larger samples than the one used here would be recommended, possibly based on a consortium collaboration favoring generalizability and cross-cultural applicability^63^ (on this regard, see the recently established “Discourse in Psychosis (DISCOURSE)” consortium: https://discourseinpsychosis.org/). Also, we still need longitudinal observations to assess the stability of the linguistic profiles and their long-term applicability for monitoring and prognostic purposes. This represents the ultimate application of these methods, consistently with a growing body of evidence showing the viability of longitudinal applications of machine-learning methods for ambulatorial and remote neuropsychological testing^63,64^.

Second, there are several limitations connected to the linguistic task. Even though the semi-structured autobiographical interview used here can be easily administered, more ecological (e.g., based on spontaneous conversations)^65^ and longer tasks (e.g., not limited to 5–6 minutes) could be more effective to capture the participants’ usual mode of communication. Considering the practical difficulties connected with the clinical application of such tasks^66^, a possible way forward could be the combination of traditional speech elicitation tasks with corpus-based approaches^67^. The use of different speech elicitation tasks, capable of triggering a broader variety of linguistic uses, could also be important. For instance, Hong et al.^21^ reported a marked difference between individuals with schizophrenia and controls in a task eliciting autobiographical experiences of five different emotions, suggesting that tasks with a higher emotional valence than the one employed here might increase the possibility of distinguishing between groups. Finally, while we analyzed language from speech to semantic classes, a more comprehensive language profiling could come from the inclusion of higher-order levels of language processing, namely discourse and pragmatic aspects, which are known for being impaired in schizophrenia^1,10^.

Taken together, our results not only confirm but also expand previous evidence of a connection between language, psychopathology, and functioning in schizophrenia, showing that a multi-layered linguistic analysis combined with clustering techniques is able to predict a wider range of symptoms (including not only negative but also positive, general, and disorganized symptoms) and several domains of daily functioning. Specifically, the outcome of our analysis shows that specific patterns of linguistic impairment (e.g., dysfluencies, underuse of pronouns and emotional and metacognitive words, etc.) underlie complex clinical states and might have a strong negative impact on participants’ functional outcomes. These findings suggest that a comprehensive multidimensional exploration of language might be useful to capture the clinical heterogeneity of schizophrenia and might have a significant clinical impact on the development of rehabilitative intervention strategies and on the monitoring of the illness course.

## Methods

### Sample

Sixty-seven individuals with a diagnosis of schizophrenia based on DSM-5 criteria^68^ were recruited from the Department of Clinical Neurosciences, IRCCS San Raffaele Scientific Institute, Milan, Italy. All participants were Italian native speakers. Exclusion criteria were: severe traumatic brain injury or neurological disorders, intellectual disability, alcohol or substance abuse in the preceding 6 months, and severe psychotic exacerbation in the preceding 3 months. All participants provided informed consent. The study was approved by the local ethical committee, following the principles of the Declaration of Helsinki.

### Assessment

Participants underwent a comprehensive assessment, including psychopathology, neurocognitive and mentalizing skills, and daily functioning. The assessment was conducted in three sessions, each lasting approximately one hour. Psychopathology was assessed by a trained psychiatrist using the PANSS^69^. A score for PANSS Positive, Negative, and General Total Scores was derived, alongside a composite score assessing the disorganization dimension^13,70^, obtained by summing the items of conceptual disorganization (P2), difficulty in abstraction (N5), stereotyped thinking (N7), mannerism (G5), disorientation (G10), poor attention (G11), lack of judgment and insight (G12), and disturbance of volition (G13). Neurocognitive abilities were assessed through the Italian version^71^ of the BACS^72^, which includes six subtests assessing Verbal Memory (VM), Digit Sequencing (DS), Token Motor Task (TMT), Semantic Fluency (SF), Symbol Coding (SC), and Tower of London (ToL). A subscore for each subtest was derived (adjusted for demographic variables), as well as an equivalent total BACS score. Sociocognitive skills were assessed via the ToM PST^73^, which includes a non-verbal ToM Sequencing Task and a ToM Questionnaire. Subscores for the Sequencing Task and the Questionnaire and a total score for global ToM abilities were derived. Finally, functioning was measured using the QLS^74^, from which a subscore for each of the three subscales (i.e., Interpersonal Relations, Instrumental Role, and Personal Autonomy) and a total score were calculated. Neurocognition, social cognition, and functioning were assessed by trained clinical psychologists.

### Semi-automated linguistic analysis

To elicit speech production, participants were administered the Interview task from the Assessment of Pragmatic Abilities and Cognitive Substrates (APACS) test^75^, a validated tool developed to assess pragmatic skills (both expressive and receptive) using the original Italian version. This task, lasting at least five minutes according to the APACS manual, consists of a semi-structured interview on autobiographical topics (i.e., family, home, work, organization of the day). Speech samples were recorded using a one-channel audio-recorder oriented towards the participant. The recordings were acquired in a quiet room in a controlled laboratorial setting. The audio recordings were then converted into .wav files and imported into the PRAAT software^76^, with a standard quality of 44.10 kHz (capturing 44100 samples per second).

The total duration of the speech sample was 6 hours and 43 minutes, with a total duration of participants’ occupation floor of 4 hours and 29 minutes (the rest being occupied by the interviewer). The mean length of participants’ interviews was 6.15 ± 2.00 minutes, with a mean occupation floor of 4.24 ± 2.25 minutes per participant.

Afterwards, audio files underwent a pre-processing phase followed by the extraction of a number of linguistic features.

#### Audio file pre-processing

The audio files were first transcribed using the Computerized Language ANalysis (CLAN) software^77^ by a linguist with training in transcription. Interviewer turns, non-verbal vocalizations, and false starts were removed. Turns were manually divided following CLAN’s criteria^77^, segmenting utterances when word strings were (a) delimited by a 1-s (or longer) pause; or (b) characterized by a terminal intonational contour; or (c) identified as a complete grammatical structure. We used the PRAAT software^76^ to quantitatively check criteria (a) and (b). Another trained linguist transcribed 16 randomly-selected interviews (i.e., 25% of the entire sample) to assess the reliability of the transcription and the utterance segmentation procedures. The Intraclass correlation coefficient (ICC), based on a two-way mixed-effects model on single measures, showed that the two coders reached excellent absolute agreement on the number of words (ICC = 0.99, *p* < 0.001, 95% CI [.99, 1]) and moderate to excellent absolute agreement on the number of utterances (ICC = 0.80, *p* = 0.001, 95% CI [0.51, 0.91]).

#### Extraction of linguistic features

Linguistic features included nine measures across four different dimensions: (1) Lexical Richness (i.e., mean lexical frequency and type-token ratio), (2) Fluency (i.e., mean length of utterance, mean gap duration, mean silent and filled pause duration, and pause-to-word ratio), (3) Frequency of Personal Pronouns, and (4) Frequency of words belonging to the Psychological Lexicon (i.e., affective words and words related to cognitive mechanisms).

For the Lexical Richness dimension, lexical frequency values for each word in the transcripts were automatically extracted from the Corpus and Frequency Lexicon of Written Italian (CoLFIS)^78^ using the R Studio software (v. 1.3.1093)^79^ to compute the mean lexical frequency, while the type-token ratio was computed on the transcripts using Python’s Natural Language Toolkit (NLTK)^80^. As for the Fluency measures, we used PRAAT’s silence detection to automatically extract the number and the duration of silences (i.e., gaps at the beginning of turns and intra-turn pauses) from the audio files; the PRAAT software was used also to manually check the number and the duration of filled pauses, in order to determine the mean pause duration and the pause-to-word ratio. Then, we computed the mean length of utterance from the transcripts using the built-in “Words per sentence” function of the Linguistic Inquiry Word Count software (LIWC2015, v. 1.6.0)^32^, based on the utterances segmented following CLAN criteria^77^. Finally, the frequency values (in percentage) of Personal Pronouns and words expressing affective content and cognitive mechanisms (here grouped under the label of Psychological Lexicon)^81^ were extracted with the LIWC2015 software^32^ (using the Italian dictionary)^82^, which automatically analyzes raw texts word-by-word, counting the occurrences of each token included in the different lexical categories.

### Statistical analysis

The statistical analysis followed three stages: (1) we first performed a Principal Component Analysis (PCA) on automatically extracted linguistic features to identify language-based principal components (PCs); (2) then, we used the PC scores to perform a data-driven cluster analysis to identify clusters of participants; (3) finally, the clusters identified by the cluster analysis were compared for demographics, psychopathology, neurocognition, social cognition, and functioning.

As for the first stage of the analysis, we performed a Principal Component Analysis (PCA) with varimax rotation on the standardized (i.e., *z*-centered) linguistic features obtained from the semi-automated linguistic analysis. We then selected the Principal Components (PCs) with eigenvalues greater than 1^83^.

Afterwards, in order to identify subgroups of participants from the linguistic-based PCs (second stage of analysis), we performed the cluster analysis using the PC scores to feed a k-means algorithm, an unsupervised machine-learning clustering algorithm. The number of centers was determined using the silhouette method^84^ starting from 25 random centroids. We validated the final cluster solution with a Linear Discriminant Analysis (LDA), based on different replications with random-split samples^55^, cross-validated with a leave-one-out procedure. The random-split samples procedure randomly assigns a subset of the participants from the original sample to either a training or a testing subset, which are used to train and test the classification performance of the algorithm. Accordingly, we randomly assigned 75%, 50%, and 25% of the participants to the training subset (and 25%, 50%, and 75% of the participants to the testing subset, respectively), to evaluate the validation results over multiple rounds with progressively decreasing training observations. To test the robustness of the obtained measure, we iterated the procedure with a standard number of iterations of random samplings (i.e., *n* = 50, as in Hong et al.^85^) for each training-testing subset (75%-25%; 50%-50%; 25%-75%). The average performance (in proportion: 0–1) of the algorithm in both training and testing partitions is then used as a measure of classification quality. Moreover, the random validation procedure was cross-validated with the leave-one-out method, which tests the classification function using all but one participant of the sample, predicting the omitted participant’s cluster membership. Therefore, the cross-validation procedure was iterated once for each participant in our dataset, using all other participants as a trainining set and the selected participant as a single-item testing set.

As for the third stage of the analysis, we compared the clusters identified by the machine-driven algorithm on demographic, clinical, cognitive, sociocognitive, and functional variables via a series of *t*-tests. Prior to the analysis, normality assumption was checked by visual inspection of the distribution of the dependent variables, while homoskedasticity assumption was assessed using the *F*-test comparing two variances (tested with the var.test function in R). When appropriate, *p*-values of pairwise comparisons were FDR-adjusted. An additional analysis was conducted to explore in a more fine-grained fashion the associations between cognitive (i.e., BACS Verbal Memory, Digit Sequencing, Token Motor Task, Semantic Fluency, Symbol Coding, and Tower of London) and sociocognitive (i.e, ToM PST Sequencing Task and Questionnaire) subscores and the linguistic-based PCs across clusters. All statistical analysis were run in R, v. 4.0.3^79^, with the R Studio editor, v. 1.3.1093.

## Supplementary information

Supplementary information for the article “Deconstructing heterogeneity in schizophrenia through language: a semi-automated linguistic analysis and data-driven clustering approach”

## Data Availability

The data are not publicly available due to restrictions, as they contain information that could compromise the privacy of research participants. The data that support the findings of this study may be available on request from the corresponding author, upon case-by-case evaluation.
