# Supplementary material for: Deconstructing heterogeneity in schizophrenia through language: a semi-automated linguistic analysis and data-driven clustering approach
Source: Schizophrenia (Heidelb). 2022 Nov 29;8(1):102. doi: 10.1038/s41537-022-00306-z (PMC9708845; doi:10.1038/s41537-022-00306-z)
Supplement: Supplementary file 1 — Supplementary information for the article “Deconstructing heterogeneity in schizophrenia through language: a semi-automated linguistic analysis and data-driven clustering approach” [file 41537_2022_306_MOESM1_ESM.docx]

**Supplementary information for the article “*Deconstructing heterogeneity in schizophrenia through language: a semi-automated linguistic analysis and data-driven clustering approach*”**

Valentina Bambini, Federico Frau, Luca Bischetti, Federica Cuoco, Margherita Bechi, Mariachiara Buonocore, Giulia Agostoni, Ilaria Ferri, Jacopo Sapienza, Francesca Martini, Marco Spangaro, Giorgia Bigai, Federica Cocchi, Roberto Cavallaro, Marta Bosia

This file contains two sections:

Section 1: additional information concerning the cluster analysis and its validation with the linear discriminant function analysis (Leave-One-Out Cross-Validation).

Section 2: Examples of speech samples elicited from two participants belonging to Cluster 1 and Cluster 2.

**Section 1.**

Supplementary Table 1. Average silhouette width for k-means algorithm, computed with an increasing number of clusters (*k*) up to 10 clusters. The optimal number of clusters is equal to the computation with highest average silhouette width (in our case, *k* = 2).

| **Average silhouette width** | **Number of clusters** | | | | | | | | |
| --- | --- | --- | --- | --- | --- | --- | --- | --- | --- |
|  | ***k* = 2** | ***k* = 3** | ***k* = 4** | ***k* = 5** | ***k* = 6** | ***k* = 7** | ***k* = 8** | ***k* = 9** | ***k* = 10** |
|  | .46 | .33 | .27 | .25 | .22 | .25 | .26 | .24 | .20 |

Supplementary Table 2. Clusters’ K-means Centroids (*z*-centered) for each principal component (PC) used in the k-means algorithm.

| **Clusters** | **PC 1**  **Lexical Richness & Fluency** | **PC 2**  **Fluency** | **PC 3**  **Frequency of Pronouns** | **PC 4**  **Frequency of Psychological Lexicon** |
| --- | --- | --- | --- | --- |
| Cluster 1 | 1.58 | .68 | .31 | -.76 |
| Cluster 2 | -3.70 | -1.59 | -0.72 | 1.79 |

Supplementary Table 3. Leave-One-Out Cross-Validation results.

| **Original**  **groups** | **Predicted groups** | | | | |
| --- | --- | --- | --- | --- | --- |
|  | **Group 1** | **Group 2** | | **Total** | |
| Group 1 | 47 (100%) | | 0 | | 47 (100%) |
| Group 2 | 4 (20%) | | 16 (80%) | | 20 (100%) |

**Section 2.**

This section includes examples of speech samples from the semi-structured interviews of two participants, one (Participant A) classified in Cluster 1 and one (Participant B) classified in Cluster 2. The two participants are close to clusters’ k-means centroids, in order to be representative of the two linguistic profiles identified by the clustering algorithm.

The excerpts of the interviews are extracted from the Interview task of the Assessment of Pragmatic Abilities and Cognitive Substrates (APACS) Test and refer to the topic “hobbies” within the interview. The speech samples were originally transcribed using CHILDES-CHAT conventions, here simplified to improve readability. Original Italian texts were literally translated into English.

From the examples in Supplementary Table 4, one can observe that Participant A produces longer utterances and shorter silent pauses and gaps compared to Participant B. Additionally, Participant A is more repetitive (for instance, the lemma *read* occurs six times) and uses overall more common words, even though there is a higher occurrence of psychological words (e.g., *remember*, *like*, *think*). Conversely, Participants B produces shorter utterances and longer silent pauses and gaps compared to Participant A. Moreover, despite the lower frequency of psychological terms, Participant B tends to use more unique and unfrequent words (e.g., *wisdom*, *psalm*, *chronological*).

Supplementary Table 5 presents the individual values for each linguistic features extracted from the semi-automated analysis and for demographic, psychopathological, and functional characteristics. Overall, Participants B shows more severe psychopathological profile and a worse daily functioning compared to Participant A.

Supplementary Table 4. Examples of speech samples.

| **Participant A from Cluster 1** | **Participant B from Cluster 2** |
| --- | --- |
| *INV: what are you reading in this period ?  *SCZ: now I’m reading The Count of Monte Cristo (0.30) / I had I had already read it two or three times / I also saw the movie / however it is always nice to read it again (0.26) / then I also brought other books / I brought Reunion (0.35) / then another book that my cousin gave me / but now I don’t remember the title (0.35) / and Uncle Tom's Cabin / and I also brought a little book of the Gospels (0.38) because &-mh I like to read them especially the Sermon the Sermon on the Mount (0.29) / it’s beautiful / I like it / and I read it with conviction because (0.37) many teachings are written there (0.37) / and I like to read them to discover new things every day (0.50) to reflect to reflect on some sentences and to discover new things / and I'm happy / I I (0.35) even if I can’t travel with books I travel anyway / they open my mind / they make me think with my head . | *INT: do you have any hobbies ?  *SCZ: (0.79) reading .  *INT: can you tell me a little about your readings ?  *SCZ: (3.24) chronological .  *INT: for instance ?  *SCZ: (5.05) wisdom books .  *INT: books ?  *SCZ: wisdom books .  *INT: can you explain to me what they are about ?  *SCZ: (0.62) Ecclesiastes (0.89) Job (2.47) / psalms .  *INT: and do you read a lot ?  *SCZ: (0.85) no .  *INT: when do you usually read ?  *SCZ: (1.26) &-eh when it happens . |
| *Note*: Utterance delimitations are indicated with the slash (/). Silent pauses and gaps are indicated between brackets. Filled pauses are preceded by the symbol “&-”.  *INT* = interviewer  *SCZ* = participant with schizophrenia | |

Supplementary Table 5. Individual values for each linguistic variable included in the multi-level linguistic analysis for Participant A (belonging to Cluster 1) and Participant B (belonging to Cluster 2), alongside demographic, psychopathological, and functional characteristics.

| **PC** | **Linguistic features** | **Cluster 1:**  **Participant A** | **Cluster 2:**  **Participant B** |
| --- | --- | --- | --- |
| **1** | **Type-token ratio** | 0.40  (404 types / 1007 tokens) | 0.69 (65 types / 94 tokens) |
|  | **Lexical frequency** | 13 812.26 (mean)  7.14 (log) | 8 769.44 (mean)  5.82 (log) |
|  | **Mean length of utterance** | 9.26 (words) | 2.07 (words) |
|  | **Gap duration (mean)** | 0.00 sec | 1.50 sec |
|  | **Pause duration (mean)*** | 0.43 sec | 1.09 sec |
| **2** | **Pause duration (mean)*** | 0.43 sec | 1.09 sec |
|  | **Pause-to-word ratio** | 0.09  (94 pauses / 1009 words) | 0.16  (15 pauses / 93 words) |
| **3** | **Frequency of personal pronouns** | 16.06% | 12.90% |
| **4** | **Frequency of affective words** | 5.05% | 0.00% |
|  | **Frequency of words related to cognitive mechanisms** | 6.44% | 2.15% |
|  | **Age (years)** | 36 | 42 |
|  | **Education (years)** | 13 | 13 |
|  | **Illness duration (years)** | 11 | 19 |
|  | **Age of onset (years)** | 25 | 23 |
|  | **PANSS Positive Scale** | 18 | 28 |
|  | **PANSS Negative Scale** | 20 | 28 |
|  | **PANSS General Scale** | 29 | 53 |
|  | **PANSS Disorganization** | 16 | 29 |
|  | **QLS Interpersonal Relations** | 15 | 11 |
|  | **QLS Instrumental Role** | 0 | 0 |
|  | **QLS Personal Autonomy** | 29 | 13 |
|  | **QLS Total Score** | 44 | 24 |
| *Note*: The individual values refer to the entire interview. PANSS = Positive and Negative Syndrome Scale for Schizophrenia; QLS = Quality of Life Scale.  * This variable is repeated in two different rows since it loads on both PC1 and PC2. | | | |
